# Oral impacts on quality of life and problem-oriented attendance among South East London adults

**DOI:** 10.1186/s12955-017-0663-3

**Published:** 2017-04-26

**Authors:** Piyada Gaewkhiew, Eduardo Bernabé, Jennifer E. Gallagher, Charlotte Klass, Elsa K. Delgado-Angulo

**Affiliations:** 1 Division of Population and Patient Health, King’s College London Dental Institute at Guy’s, King’s College and St. Thomas’ Hospitals, Denmark Hill Campus, Bessemer Road, London, SE5 9RS UK; 2 Department of Community Dentistry, Faculty of Dentistry, Mahidol University, Bangkok, Thailand; 3 Public Health England - London Region, London, UK; 4 Departamento Académico de Odontología Social, Universidad Peruana Cayetano Heredia, Lima, Peru

**Keywords:** Quality of Life, Oral Health, Dental Health Services/utilization, Adults

## Abstract

**Background:**

Dental care seeking behaviour is often driven by symptoms. The value of oral health related quality of life (OHRQoL) measures to predict utilisation of dental services is unknown. This study aims to explore the association between OHRQoL and problem-oriented dental attendance among adults.

**Methods:**

We analysed cross-sectional data for 705 adults, aged 16 years and above, living in three boroughs of Inner South East London. Data were collected during structured interviews at home. The short form of the Oral Health Impact Profile (OHIP-14) was used to assess the frequency of oral impacts on daily life in the last year. Problem-oriented attendance was defined based on time elapsed since last visit (last 6 months) and reason for that visit (trouble with teeth). The association between OHIP-14 (total and domain) scores and problem-oriented attendance was tested in logistic regression models adjusting for participants’ sociodemographic characteristics.

**Results:**

Problem-oriented attenders had a higher OHIP-14 total score than regular attenders (6.73 and 3.73, respectively). In regression models, there was a positive association between OHIP-14 total score and problem-oriented attendance. The odds of visiting the dentist for trouble with teeth were 1.07 greater (95% Confidence Interval: 1.04–1.10) per unit increase in the OHIP-14 total score, after adjustment for participants’ sociodemographic characteristics. In subsequent analysis by OHIP-14 domains, greater scores in all domains but handicap were significantly associated with problem-oriented attendance.

**Conclusion:**

This study shows that oral impacts on quality of life are associated with recent problem-oriented dental attendance among London adults. Six of the seven domains in the OHIP-14 questionnaire were also associated with dental visits for trouble with teeth.

## Background

Dental care-seeking behaviour is mainly motivated by symptoms [1, 2]. The Behavioural Model of Health Services Use [3, 4] has been used to understand how contextual- and individual-level characteristics influence health services utilisation, including dental services use [5–7]. According to the model, individuals will seek health care only when they perceive a need for treatment [3, 4]. People’s perception of their oral health status and the related oral impacts on daily life are important in planning services designed to improve their quality of life [8]. From this point of view, oral health-related quality of life (OHRQoL) measures might be useful for the identification of people with the greatest need and priority for care. However, not everybody having oral impacts on daily activities may demand dental care. Therefore, the potential of OHRQoL measures to predict the use of dental services is still unknown.

Earlier studies on the relationship between OHRQoL and dental services utilisation were conducted in children [9–11]. While two of those studies showed the impact of oral conditions on children’s quality of life was positively associated with recent visits to the dentist [9, 10], the remaining study reported no association between the child’s quality of life and utilisation of dental services [11]. What is more, the authors also showed that oral impacts on the family (parental distress and family function) were associated with lower utilisation of dental services [11]. The few studies in adults also showed conflicting results. A study among older adults in Sendai (Japan) reported no significant association between impaired OHRQoL, as measured by the Oral Impacts on Daily Performances (OIDP) instrument, and dental services use within the past year in crude or adjusted regression models [12]. A study among Canadian adults showed that having difficulties in chewing and sleep due to oral conditions were significantly associated with problem-oriented visits to the dentist among the working poor (i.e., working people with incomes below a certain poverty threshold). These associations were attenuated after controlling for participants’ socio-demographic characteristics. In addition, the presence of pain/discomfort, feelings of embarrassment and difficulty in working due to oral conditions were not significantly associated with problem-oriented attendance [13]. Lastly, a cross-sectional study among 344 underprivileged older people living in Jerusalem (Israel) showed no significant association between the short form of the Oral Health Impact Profile (OHIP-14) and dental visiting in the last 6 months after adjustments for socio-demographic factors [14]. Further research using population-based samples, as opposed to specific population sub-groups (such as the working poor or older adults) and carried out in alternative settings is needed to shed some lights on the hypothesised association. Such information would support the use of OHRQoL measures for planning dental services and predicting utilisation. A study was set out to determine the association between OHRQoL and problem-oriented dental attendance among adults. It was hypothesised that adults who experienced oral impacts on daily life were more likely to visit the dentist for trouble with teeth than those who did not experience such impacts.

## Methods

### Data source

A population-based oral health survey was carried out in Inner South East London to identify perceived dental needed and dental care seeking behaviour of adults in Inner South East London. King’s College London Research Ethics Committee approved the survey protocol. A representative, multi-ethnic sample of the general population aged 16 years and above living in Lambeth, Southwark and Lewisham (approx. 580,000 inhabitants) was recruited using stratified multistage random sampling. A list of all valid addresses for postal delivery in each borough (stratum) organised by postcode sectors (primary sampling units) was the sampling frame. Addresses were selected in two stages. Fifteen postcode sectors were first selected from each borough randomly and a sample of addresses was subsequently selected from each sector randomly. Household rosters were generated in every eligible household, from which only one adult was invited to participate. A target of 500 addresses per borough was initially set out (1500 overall, sampling fraction: 0.003). Of them, 1244 were valid and 770 (62% response rate) agreed to take part in the survey [15]. Based on the valid addresses, no contact was possible with 25% of households (after 4 calls were made to the address) and a further 13% of households refused to participate in the survey.

For the present analysis, 65 participants were excluded because of missing values on ethnicity (*n* = 32), OHRQoL (*n* = 22) and social grade (*n* = 14). Our study sample included 695 adults (56% of the eligible sample). A power calculation showed that our analytical sample had 90% power to detect a difference in the OHIP-14 total score of 2 units or greater between regular and problem-oriented attenders (using a 5 to 1 ratio), at 5% significance level and assuming a common standard deviation of 6 units.

### Data collection

Data were collected through structured interviews at home. All fieldwork was carried out by 12 experienced and trained interviewers. The interviewer made arrangements to return at a later date with a translator if needed. The survey instrument gathered information on sociodemographic characteristics (sex, age and ethnicity), dental care seeking behaviour and perceived dental needs, all based on previous national surveys [16–18]. Participants ethnicity was self-assigned as White, Asian, Black, Mixed and Other [18]. Participants’ socioeconomic status was determined by the social grade of the chief income earner (CIE) in the household. Information on the CIE current job or occupation, employment status, size of organization and supervisory status were used to classify them as: high managerial, administrative, or professional (A); intermediate managerial, administrative, or professional (B); supervisory, clerical, and junior managerial, administrative, or professional (C1); skilled manual workers (C2); semi- and unskilled manual workers (D); and state pensioners, casual, or lowest-grade workers, or unemployed with state benefits only (E) [19]. For analysis, three groups were generated by merging grades A and B (highest group), C1 and C2 (intermediate), and D and E (lowest) [15, 20].

Participants’ OHRQoL was assessed with the short form of the Oral Health Impact Profile (OHIP-14) [21], which measures the frequency of oral impacts on everyday life within the past year. Questionnaire items are organised in 7 domains: functional limitation, physical pain, psychological discomfort, physical disability, psychological disability, social disability and handicap. Responses were provided using 5-point ordinal scales (never = 0, hardly ever = 1, occasionally = 2, fairly often = 3 and very often = 4). Domain scores were calculated by adding the responses to the two corresponding items (range: 0 to 8) and the total score by adding the responses to all 14 items (range: 0 to 56). Higher scores indicated worse OHRQoL [22, 23]. Participants with missing values in one or more OHIP-14 items were excluded [24, 25].

Participants also reported the time since last dental visit (≤6 months, between 7 and 12 months, between 13 and 24 months, over 2 years ago and never been) and the reason for that visit (trouble with teeth, check-up and other reasons). Based on the answers to both questions, recent problem-oriented attendance was defined as having visited the dentist in the past 6 months for trouble with teeth. The 6-month threshold was selected to mimic a temporal ordering between exposure (oral impacts) and outcome (dental attendance) and identify dental services use that most likely occurred after the experience of oral impacts [10].

### Statistical analysis

All analyses were weighted to correct for differences in the probability of selection because of non-response and non-coverage, and to adjust for differences in the age-by-sex-by-ethnicity distribution between the sample and the general adult population 16 years or older in the three London boroughs included in the study, according to the 2001 UK Census [15, 20].

We first present the characteristics of the sample and compare them against those excluded due to missing values with the Chi-square test to evaluate the impact of missing data. We then compared the proportion of problem-oriented attenders by sociodemographic characteristics with the Chi-square test. OHIP-14 total and domain scores were compared between problem-oriented and regular attenders using the *t*-test. The association between OHIP-14 total score and recent problem-oriented attendance was assessed in crude and adjusted logistic regression models. The adjusted model controlled for sex, age groups, ethnicity, borough of residence and social grade. A similar set of models was used when testing the association between each OHIP-14 domain score and problem-oriented attendance. Multilevel modelling (participants nested within boroughs) was not considered due to the limited number of clusters. Instead, we included borough of residence as an explanatory variable in regression models as it is often done for geographical variables with few groupings such as rural/urban status.

## Results

The study sample consisted of 705 adults (51% women) residing in Inner South East London. Table 1 shows the sociodemographic characteristics of the study sample. There were no significant differences in sociodemographic composition, OHIP-14 scores and dental attendance pattern between the study sample and those excluded because of missing data on relevant variables. The proportion of problem-oriented attenders was 15.4%, with no differences by sociodemographic factors.

**Table 1:** Problem-oriented attendance among adults in South East London, according to socio-demographic characteristics

Characteristics	All sample		Problem-oriented attenders		
	n^a^	%	n^a^	%	*P* value^b^
*Sex*					
Men	312	49.0	44	14.5	0.486
Women	393	51.0	64	16.3	
*Age group*					
16–24 years	69	14.0	9	13.1	0.096
25–34 years	145	29.4	26	17.7	
35–44 years	191	21.4	25	12.6	
45–54 years	98	11.7	21	21.4	
55–64 years	82	10.1	18	19.7	
65–74 years	66	7.5	8	14.8	
75 or more	54	5.8	1	2.4	
*Ethnicity*					
White	478	72.0	70	14.9	0.776
Black	193	22.8	31	16.1	
Asian	34	5.3	7	18.9	
*Social grade*					
High	125	18.5	16	13.7	0.774
Medium	282	42.4	48	16.4	
Low	298	39.1	44	15.2	
*Borough*					
Lambeth	228	33.7	29	12.1	0.205
Southwark	236	32.9	39	16.4	
Lewisham	241	33.4	40	17.8	

^a^ Counts are unweighted

^b^ Chi-square test was used for comparison

The mean OHIP-14 score was 4.19 (SD: 6.06; range: 0 to 50), with the highest score for physical pain (mean: 1.50, SD: 1.66) and the lowest score for social disability (mean: 0.22 SD: 0.77). Problem-oriented attenders had a significantly higher OHIP-14 total score than regular attenders (6.73 versus 3.73). Problem-oriented attenders also had significantly higher OHIP-14 scores than regular attenders in all domains except for handicap (Table 2). The largest differences were found in physical pain (2.18 versus 1.38) and physical disability (0.86 versus 0.27), whereas the smallest (although significant) difference was found in psychological disability (0.79 versus 0.53).

**Table 2:** Comparison of OHIP-14 domain and total scores between problem-oriented and regular attenders in South East London

OHIP-14 domains	Regular attenders (*n* = 597)		Problem-oriented attenders (*n* = 108)		*P* value^a^
	Mean	(SD)	Mean	(SD)	
Functional Limitation	0.41	(0.98)	0.75	(1.26)	0.009
Physical Pain	1.38	(1.61)	2.18	(1.80)	<0.001
Psychological Discomfort	0.69	(1.37)	1.22	(1.82)	0.005
Physical Disability	0.27	(0.73)	0.86	(1.54)	<0.001
Psychological Disability	0.53	(1.13)	0.79	(1.28)	0.046
Social Disability	0.25	(0.77)	0.59	(1.19)	0.005
Handicap	0.20	(0.73)	0.34	(0.99)	0.135
Total score	3. 73	(5.61)	6.73	(7.67)	<0.001

^a^ *T*-test was used for comparison

There was a positive association between OHIP-14 total score and problem-oriented attendance (Table 3). The odds of visiting the dentist when having trouble with teeth were 1.07 greater (95% Confidence Interval: 1.04–1.10) per unit increase in the OHIP-14 total score, after adjustment for participants’ sociodemographic characteristics. In subsequent analysis by OHIP-14 domains, greater scores were significantly associated with problem-oriented attendance except for handicap. The adjusted odds of visiting the dentist when having trouble with teeth were 1.35 (95%CI: 1.12–1.61), 1.31 (95%CI: 1.16–1.47), 1.24 (95%CI: 1.09–1.40), 1.68 (95%CI: 1.39–2.04), 1.19 (95%CI: 1.01–1.39) and 1.38 (95%CI: 1.13–1.68) times greater per unit increase in the scores for functional limitation, physical pain, psychological discomfort, physical disability, psychological disability and social disability, respectively (Table 4).

**Table 3:** Models for the association between OHIP-14 total score and problem-oriented attendance among adults in South East London (*n* = 705)

Explanatory variables	Crude associations			Adjusted associations		
	OR^a^	[95% CI]	*P* value	OR^a^	[95% CI]	*P* value
*OHIP-14 total score*	1.07	[1.04–1.10]	<0.001	1.07	[1.04–1.10]	<0.001
*Sex*						
Men	1.00	[Reference]		1.00	[Reference]	0.667
Women	1.14	[0.76–1.72]	0.519	1.10	[0.72–1.69]	
*Age group*						
16–24 years	1.00	[Reference]		1.00	[Reference]	
25–34 years	1.41	[0.71–2.78]	0.328	1.33	[0.66–2.70]	0.428
35–44 years	0.97	[0.46–2.05]	0.926	0.78	[0.36–1.71]	0.536
45–54 years	1.75	[0.80–3.84]	0.161	1.38	[0.61–3.15]	0.442
55–64 years	1.61	[0.71–3.67]	0.255	1.37	[0.59–3.21]	0.468
65–74 years	1.09	[0.42–2.86]	0.858	0.83	[0.30–2.26]	0.713
75 or more	0.13	[0.01–1.29]	0.082	0.12	[0.01–1.15]	0.066
*Ethnicity*						
White	1.00	[Reference]		1.00	[Reference]	
Black	1.08	[0.66–1.76]	0.755	1.02	[0.60–1.71]	0.954
Asian	1.37	[0.59–3.20]	0.464	1.34	[0.56–3.21]	0.510
*Social grade*						
High	1.00	[Reference]		1.00	[Reference]	
Medium	1.25	[0.69–2.23]	0.462	0.28	[0.76–2.58]	0.278
Low	1.12	[0.62–2.04]	0.701	0.44	[0.68–2.44]	0.440
*Borough*						
Lambeth	1.00	[Reference]		1.00	[Reference]	
Southwark	1.44	[0.86–2.43]	0.168	0.27	[0.79–2.32]	0.267
Lewisham	1.59	[0.95–2.66]	0.075	0.06	[0.98–2.85]	0.060

^a^ Logistic regression was fitted and odds ratios (OR) reported. The outcome measure was problem-oriented attendance. The adjusted model includes all factors listed in the table as explanatory variables

**Table 4:** Models for the association between OHIP-14 domain scores and problem-oriented attendance among adults in South East London (*n* = 705)

OHIP-14 domain	Unadjusted associations			Adjusted associations		
	OR^a^	[95% CI]	*P* value	OR^a^	[95% CI]	*P* value
Functional Limitation	1.29	[1.10–1.52]	0.002	1.35	[1.12–1.61]	0.001
Physical Pain	1.30	[1.16–1.46]	<0.001	1.31	[1.16–1.47]	<0.001
Psychological Discomfort	1.23	[1.09–1.38]	0.001	1.24	[1.09–1.40]	0.001
Physical Disability	1.65	[1.38–1.98]	<0.001	1.68	[1.39–2.04]	<0.001
Psychological disability	1.18	[1.02–1.38]	0.032	1.19	[1.01–1.39]	0.036
Social disability	1.42	[1.17–1.72]	<0.001	1.38	[1.13–1.68]	0.001
Handicap	1.22	[0.98–1.52]	0.072	1.20	[0.96–1.50]	0.108

^a^ Logistic regression was fitted and odds ratios (OR) reported. All models were adjusted for sex, age groups, ethnicity, borough of residence and social grade

## Discussion

This study shows that adults with poorer oral health related quality of life in the past year were more likely to have a recent visit to the dentist for trouble with their teeth. In addition, greater scores in all OHIP-14 domains in OHIP-14, except for handicap, were significantly associated with problem-oriented attendance. These associations were robust to adjustments for participants’ sociodemographic characteristics.

Some study limitations need to be considered. First, analyses were based on cross-sectional data and thus restricted to identify associations between the variables of interest rather than causal relationships. This is a relevant issue as it is not possible not know if oral impacts led to dental services use or vice-versa. However, we attempted to minimise this issue by using a longer recall period for oral impacts than for dental services utilisation (12 and 6 months, respectively). Second, the study sample represented 56% of the eligible sample, which some may view as non-representative. However, we used analytical weights to correct for key demographic differences between the study sample and the local population living in Inner South East London, thus allowing for the generalisation of findings to the study population. That said, the present findings are not generalizable beyond the study population (external validity). Third, participants’ utilisation of dental services was obtained through self-reports, which could be affected by information bias. However, self-reported dental visits have shown high levels of agreement with data from dental records [26, 27]. Fourth, we could not adjust for clinical measures of oral health (dental caries, periodontal disease, number of teeth) because these data were not collected in the survey. Clinical oral health may confound the association between OHRQoL and dental services utilisation. We attempted to minimise this effect by controlling for more distal determinants of oral health, such as social grade and ethnicity, because poor oral health is overrepresented among ethnic minorities [28, 29] and lower socioeconomic groups [30].

Going back to our findings, the magnitude of the association between oral impacts on quality of life and problem-oriented attendance was such that the odds of visiting the dentist for trouble with teeth increased by 7% per every unit increase in the OHIP-14 total score. There was a clear dose-response relationship, with greater odds of using dental services at higher OHIP-14 scores. Our finding disagrees with the only previous study that used the OHIP-14 to measure OHRQoL [14]. This difference could be explained by the sample composition (underprivileged older adults with similar levels of oral impacts), the definition of dental attendance used (any visit to a specific hospital in the last 6 months) and the higher levels of oral impacts on quality of life (mean OHIP-14 score: 10.43) found in the previous study.

Six of the seven domains in Locker’s model of consequences of oral health [31], spanning from functional limitation to physical pain to discomfort to disability (physical, psychological and social disability), were positively associated with problem-oriented attendance. Interestingly, it was not physical pain but physical disability the domain most strongly associated with problem-oriented attendance. Difficulty in eating among children [10] and difficulty in chewing among adults (although not significantly in multivariable regression models) [13] have been previously reported as stronger indicators of utilisation of dental services. The two items in the physical disability domain were ‘have you had to interrupt meals’ and ‘has your diet been unsatisfactory because of problems with your teeth, mouth or dentures’. Given the fact that the item ‘have you found it uncomfortable to eat any foods’ was included in the physical pain domain, the current findings suggest that it is the enjoyment of food rather than merely chewing which may trigger a dental visit. On the other hand, handicap was the only domain not significantly associated with problem-oriented attendance. This finding may be explained by the broader items included in this domain (‘have you felt that life in general was less satisfying’ and ‘have you been totally unable to function’), which did not emphasise on specific signs and symptoms related to dental problems. The lower frequency of oral impacts at handicap level may also explain the negative findings. Indeed, fewer impacts on the handicap domain of the OHIP-14 have been reported in the last two Adult Dental Health Surveys in the UK [32, 33].

There are some implications for policy and research. There might be some benefit in using OHRQoL measures for planning and predicting utilisation of dental services. In this regard, the socio-dental approach is a way forward in the complementary use of clinical and perceived measures for needs assessment, planning and prioritisation of services [34, 35]. Information on the association between OHRQoL measures and use of dental services is scarce. Further research is thus needed, using longitudinal data, from alternative settings and assessing not only the frequency but also the severity of oral impacts in relation to accessing dental services in order to understand if oral impacts on quality of life could predict dental services utilisation. It would also be interesting to know whether normative or perceived need (of which OHRQoL are only an indicator) is the strongest predictor of utilisation of dental services.

## Conclusions

This study shows that oral impacts on quality of life in the last year are associated with recent problem-oriented dental attendance among adults in Inner South East London. Six of the seven domains in the OHIP-14 questionnaire were also associated with dental visits for trouble with teeth.

## Abbreviations

CI: Confidence Interval
OHIP: Oral Health Impact Profile
OHRQoL: Oral health-related quality of life
OIDP: Oral Impacts on Daily Performances
OR: Odds ratios
SD: Standard Deviation
UK: United Kingdom
